# Climate Change and Water Use Partitioning by Different Plant Functional Groups in a Grassland on the Tibetan Plateau

**DOI:** 10.1371/journal.pone.0075503

**Published:** 2013-09-17

**Authors:** Jia Hu, Kelly A. Hopping, Joseph K. Bump, Sichang Kang, Julia A. Klein

**Affiliations:** 1 Ecology Department, Montana State University, Bozeman, Montana, United States of America; 2 Graduate Degree Program in Ecology, Colorado State University, Colorado, United States of America; 3 School of Forest Resources & Environmental Science, Michigan Technological University, Michigan, United States of America,; 4 Institute of Tibetan Plateau Research, Chinese Academy of Sciences, Beijing, China; 5 Department of Ecosystem Science & Sustainability, Colorado State University, Colorado, United States of America; Tennessee State University, United States of America

## Abstract

The Tibetan Plateau (TP) is predicted to experience increases in air temperature, increases in snowfall, and decreases in monsoon rains; however, there is currently a paucity of data that examine the ecological responses to such climate changes. In this study, we examined the effects of increased air temperature and snowfall on: 1) water use partitioning by different plant functional groups, and 2) ecosystem CO_2_ fluxes throughout the growing season. At the individual plant scale, we used stable hydrogen isotopes (δD) to partition water use between shallow- and deep-rooted species. Prior to the arrival of summer precipitation (typically mid-July), snowmelt was the main water source in the soils. During this time, shallow and deep-rooted species partitioned water use by accessing water from shallow and deep soils, respectively. However, once the monsoon rains arrived, all plants used rainwater from the upper soils as the main water source. Snow addition did not result in increased snowmelt use throughout the growing season; instead, snowmelt water was pushed down into deeper soils when the rains arrived. At the larger plot scale, CO_2_ flux measurements demonstrated that rain was the main driver for net ecosystem productivity (NEP). NEP rates were low during June and July and reached a maximum during the monsoon season in August. Warming decreased NEP through a reduction in gross primary productivity (GPP), and snow additions did not mitigate the negative effects of warming by increasing NEP or GPP. Both the isotope and CO_2_ flux results suggest that rain drives productivity in the Nam Tso region on the TP. This also suggests that the effects of warming-induced drought on the TP may not be mitigated by increased snowfall. Further decreases in summer monsoon rains may affect ecosystem productivity, with large implications for livestock-based livelihoods.

## Introduction

Over the past fifty years, the Tibetan Plateau (TP) has experienced climate warming at a rate of 0.25°C per decade [1]. This increase is twice the mean global warming rate and has the potential to affect regional hydrology, ecosystem productivity, and shifts in species composition [2]–[5]. Unlike other alpine grasslands in many parts of the world, on the TP, annual precipitation is concentrated in the summer monsoon season. The TP receives 20% of its annual precipitation during winter and 80% during the summer. During the winter, Westerlies bring moisture from the Atlantic Ocean; during the summer, the rains originate from the Indian Ocean, via the Bay of Bengal [6], [7]. The winters are characterized as dry and cold, and there is usually no consistent snowpack, although large winter/spring snowstorms do occur and can cover the vegetation for an extended period of time [8]. In addition to predicted increases in temperature, future climate models for this area are predicting an increase in snowstorms [3], [9] and decreasing monsoon precipitation [10], [11], but the impact of changes in precipitation dynamics on ecological processes, such as shifts in species composition, and nutrient and carbon dynamics remain relatively unknown.

Understanding intra- and interannual plant-moisture dynamics and how they differ by plant functional groups under predicted climate change scenarios has important implications for plant community composition, ecosystem processes, and livelihood through livestock production on the TP. For example, in the past, intense snowstorms have caused high animal mortality rates because the animals starve when they cannot access the senesced vegetation under the frozen snow [8], [12]. However, despite the negative consequences of snowstorms on animal mortality, there is no negative effect on the vegetation [8], [13]. In fact, increased snow may alleviate some of the water stress that plants experience under warmer climatic conditions. On the TP, soil moisture levels typically begin to decline from spring to mid-summer, before the monsoon rains arrive, and soil moisture does not begin to increase until after the arrival of monsoon rains [14]. Studies have also found that experimental warming causes soils to dry earlier [15]–[17]. Therefore, an increase in snow may increase soil moisture earlier in the growing season, leading to less warming-induced soil moisture depletion during mid-summer. Furthermore, the increase in soil moisture due to increased snow may benefit the shallower rooted graminoid species that dominate the grasslands by providing a water source during the pre-monsoon dry period.

At the larger, ecosystem scale, warming and increases in snowfall will also affect above and below ground carbon dynamics. Current studies suggest that alpine meadows on the TP are a carbon sink (Kato *et al*., 2004, Kato *et al*., 2006, Yu *et al*., 2012), although how carbon dynamics in the region will respond to climate change remains unknown. Past warming manipulations studies examining CO_2_ flux responses in high latitude and altitude ecosystems have found mixed results; some studies found warming to increase CO_2_ uptake in moist Arctic ecosystems, while warming increased CO_2_ efflux in drier Arctic ecosystems [18]–[20]. Other studies have also found no change in net CO_2_ flux because increased gross primary productivity was offset by increases in ecosystem respiration [20], [21]. However, the TP is unique from other arctic and alpine ecosystems because monsoon rain is the dominant water source, and this difference in precipitation regime may influence both the magnitude and timing of peak net ecosystem productivity (NEP). Furthermore, while alpine grasslands of the TP occupy 63 million ha. of landmass [22], there are currently no experiments exploring how changes in air temperature and precipitation will affect net ecosystem productivity (NEP) in this ecosystem type.

The main objective of this study was to understand how changes in winter/spring snow precipitation and climate warming affect water and carbon dynamics in this region. We address this objective at two spatial scales: the individual plant scale and the ecosystem (plot) scale. At the plant scale, we first use stable isotope analysis of hydrogen to explore: 1) changes in water source across a growing season, and 2) water partitioning by the different plant functional groups. At the plot scale, we then coupled these isotope measurements with measurements of CO_2_ flux to determine how increased air temperature and increased snow influences the timing of peak carbon uptake in an alpine ecosystem on the Tibetan Plateau.

## Materials and Methods

### Site description

The study was conducted near the Nam Tso Monitoring and Research Station (30°46′N, 90°59′E), located in the Tibetan Autonomous Region at an elevation of 4730 m. The Chinese government and the Chinese Academy of Sciences gave us permission to conduct research at the Nam Tso Research Station and the village leaders gave us permission to establish our experimental plots on their land. The field site is 230 km north of Lhasa at the base of the Nyenchentaglha Mountains, close to Nam Tso Lake. This area is summer grazing land used by semi-nomadic herders. Mean annual temperature is −0.6°C and precipitation is 414.6 mm [23]. The vegetation is characterized as alpine meadow vegetation, with the sedge, *Kobresia pygmaea* dominating the landscape.

### Experimental Design

**Figure 1:**
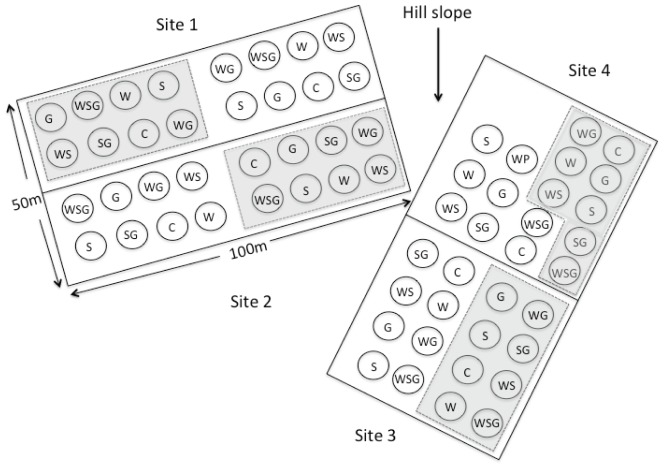
Experimental design and plot layout. The four treatments were: C = control, S = snow addition, W = warming, and G = yak grazing (not included in this study). Each site had 16 treatments: eight plots including pikas (non-shaded plots) and eight plots excluding pikas (plots shaded in gray). Each 16 treatment plots is replicated four times (4 sites). The plots were blocked horizontally for Sites 1 and 2 but vertically for Sites 3 and 4 to account for the hillside slope. Plots shaded in gray were not included in this study.

The experiment was established in 2009 and consisted of two climate change manipulations (warming and snow addition) and two grazing factors (yaks and pikas). This resulted in 16 treatment combinations that were replicated in four sites for a total of 64 plots. At each site, pikas were present in half of the plots (n = 8) and excluded from half of the plots (n = 8). For this study, we focused on the climate treatments (warming and snow addition) in plots where no current yak grazing occurred and pikas were not excluded, resulting in the following treatments: control (C), warming (W), snow addition (S), and warming×snow addition (WS) (Figure 1). All four sites shared similar soil characteristics and vegetation composition.

Snow was applied to the plots from April 26-May 3 in 2009 and April 25-April 29 in 2010. During both these snow addition periods, there was no standing snowpack at the site. Snow was transported in from nearby areas and applied onto the plots using wire mesh and tarpaulin forms to position snow additions. The resulting cylindrical snow “cakes” were approximately 1 m in diameter and 0.5 m in height. The snow water equivalent (SWE) was estimated to be 0.30 m (SWE = depth/density). Using the regional mean snow data, we estimate that the experimental snow addition was approximately equivalent to a 1.3 m snowfall; this is within the range of a large ‘snow disaster’ snowstorm [24]. The snow cakes all melted within a 6-day period in 2009 and within a 5-day period in 2010.

Warming was achieved using conical fiberglass open top chambers (OTCs) that measured 1.5 m diameter at the base, 0.75 m diameter at the top, and 0.4 m high. The International Tundra Experiment has used these OTCs extensively across the world [25]–[27]. OTCs were installed at the start of the growing season (mid-May) and removed at the end of the growing season (early September) every year.

### Microclimate measurements

We measured air temperature at 10 cm above the ground using the Decagon ECT sensor fitted with a radiation shield (Decagon Devices, Pullman, Washington, USA). We also measured soil moisture and temperature integrated over the top 10 cm using both the Decagon 5TM and EC-TM sensors. Sensors logged data from 28 June to 24 August, 2009 (n = 3 for each treatment) and from 1 May to 26 August, 2010 (n = 4 for each treatment). In 2009, the sensors were not installed in our research site until late June due to clearing the sensors through customs.

### Source water, soil, and plant water isotopes

We collected snow, rain and ground water throughout the growing season. Snow samples were collected using a large PVC pipe fitted with a closed top. An entire column of the snowpack was collected and then allowed to melt before subsamples were collected. Rain was collected using a Nalgene bottle fitted with a funnel and topped with mineral oil to prevent evaporation. Rain was collected every two weeks. Ground water was also collected every two weeks from a nearby well (approximately 100 m away).

We collected plant and soil samples to identify the sources of water utilized by plants as well as the depth from which plants obtained water. In both 2009 and 2010, we synchronized plant and soil collection with changes in precipitation dynamics. In 2009 we collected samples on 28 June (pre-monsoon), 28 July (monsoon), and 23 August (late monsoon) outside the treatment plots because we were still installing the treatments. In 2010, we collected on 8 June (pre-monsoon), 6 July (early-monsoon), 3 August (monsoon), and 26 August (late monsoon) from the following treatment plots: control (C), warming (W), snow addition (S), warming×snow addition (WS) in all four sites (n = 4). In order to minimize disturbance to the soils, we collected only one soil core from each treatment. Soil from 5–10 cm increments were placed in vials, sealed with Parafilm (Parafilm ®) and kept frozen.

In both 2009 and 2010, we collected the most common plant species from three different plant functional groups (graminoids, forbs, and shrub), and with varying rooting depths (5 to 35 cm). Rooting depth was determined by digging up the roots for all the sampled species outside of our experimental plots. We were able to collect two more species in 2009 than in 2010 because not all species were present in the treatment plots in 2010. The species collected were (in order of shallow to deep rooting depth): *Kobresia pygmaea* (graminoid), *Leontipodium. Pussilum* (forb), *Gentiana farreri* (forb, collected only in 2009), *Potentilla saundersiana* (forb), *Potentilla fruticosa* (shrub), *Androsace tapete* (forb, collected only in 2009), *Oxytropis stracheyana* (forb, collected only in 2009), and *Astragalus rigidulus* (forb, collected only in 2010). During each collection, we gently dug up each species and removed the soil from the roots. Because the root biomass for many of the plants was small, we usually combined roots from multiple plants into the same vial. The roots were then placed in a glass vial with a Teflon coated cap, sealed with Parafilm and kept frozen.

We extracted the soil and plant root water samples in the US using cryogenic distillation [28]. All water source samples (rain, snow, and ground) were analyzed for both oxygen (δ^18^O) and deuterium (δD) isotopic composition at the Center for Stable Isotope Biogeochemistry at University of California, Berkeley. Because we could not extract enough water from all of our plant samples for both oxygen and hydrogen isotope analysis, we present the data for δD only for plant and soil samples. Stable isotope ratios of hydrogen were expressed using δ notation (units of ‰), where δD = (R_sample_/R_standard_ – 1) *1000, and R_sample_ and R_standard_ are the molar ratios of D/H of the sample and standard water (VSMOW), respectively.

We also calculated deuterium excess (*d*) from the relationship between δD and δ^18^O of precipitation to understand the influence of the Indian Monsoon versus local convective storms on the rain isotopic signature. Deuterium excess was calculated as: *d* = δD – 8*δ^18^O [29].

### CO_2_ Flux measurements

Due to the small size of the vegetation at Nam Tso, we were unable to make photosynthesis measurements on individual plant species. Alternatively, we measured net ecosystem productivity (NEP) and ecosystem respiration (ER) to calculate gross primary productivity (GPP) at the plot scale. This allowed us to determine when the system became photosynthetically active and to examine the seasonal trends in carbon dynamics. NEP and ER were measured in the plots during the three periods in 2010 that correspond to pre-monsoon (10 June), pre-monsoon (3 July), and monsoon (8 August) periods, and at midday (from 11:00 to 13:00). We limited CO_2_ flux measurements during this time period because environmental conditions changed quickly on the TP and we aimed to keep light levels and air temperature relatively constant for all the plot measurements. For each measurement, one treatment plot from three different sites was measured for a total of three replicates (n = 3). We were unable to measure all treatments from all four sites (for a n = 4) due to time constraints.

We used a Li-6400 Portable Photosynthesis System (LI-COR Inc., Lincoln, Nebraska, USA) fitted with a custom chamber to measure ecosystem CO_2_ flux. The chamber was made from Lexan and measured 0.5 m×0.5 m×0.25 m. A portable base was constructed using Lexan with an opening of 0.5 m×0.5 m. Plastic sheeting was attached to the outside of the base to create a plastic skirt, and a heavy chain was used to secure the base and skirt in place [30]. This created a seal between the ground and chamber. Two small fans continuously mixed air inside the chamber during measurements.

During each measurement, the base and chamber were first placed over the plot. NEP measurements were logged after the change in CO_2_ concentration became steady [31], [32]. The plot was allowed to vent, and then two additional NEP measurements were taken in the same manner. For ER, a dark shroud was placed over the chamber, and then CO_2_ flux measurements were again taken in the same manner. This resulted in three NEP and three RE measurements per plot, which were averaged to produce one flux value. Gross Primary Productivity (GPP) was calculated as: NEP = GPP – ER [33], such that NEP is expressed as a positive value.

### Statistical Analysis

All statistical analyses were performed in SPSS (IBM SPSS Statistics Version 19). To test for differences in air temperature, soil temperature, and soil moisture among the four treatments (C, W, S, WS), we ran a one-way repeated measure ANOVA, combined with a Bonferroni post hoc test. To test for differences in plant water δD in 2009, we ran a two-way ANOVA, with species and date as factors and looked for interaction between species and date. For the 2010 plant δD data, we used a three-way ANOVA, with date, treatment, and species as factors, and looked for interactions between the three factors. A Bonferroni post hoc test was used for pairwise comparisons for plant water δD data for both 2009 and 2010. For the CO_2_ flux measurements, we used a multiple regression to evaluate the influences of soil moisture, soil temperature, and light on NEP, since all three of these environmental variables changed throughout the growing season. In order to test for differences in NEP, ER, and GPP, we ran a two-way ANOVA, with treatment and date as factors. To examine differences in treatment within each sampling period, we ran a one-way ANOVA, with treatment as the only factor. We used the least-square difference (LSD) for all pairwise treatment comparisons.

## Results

### Microclimate measurements

**Figure 2:**
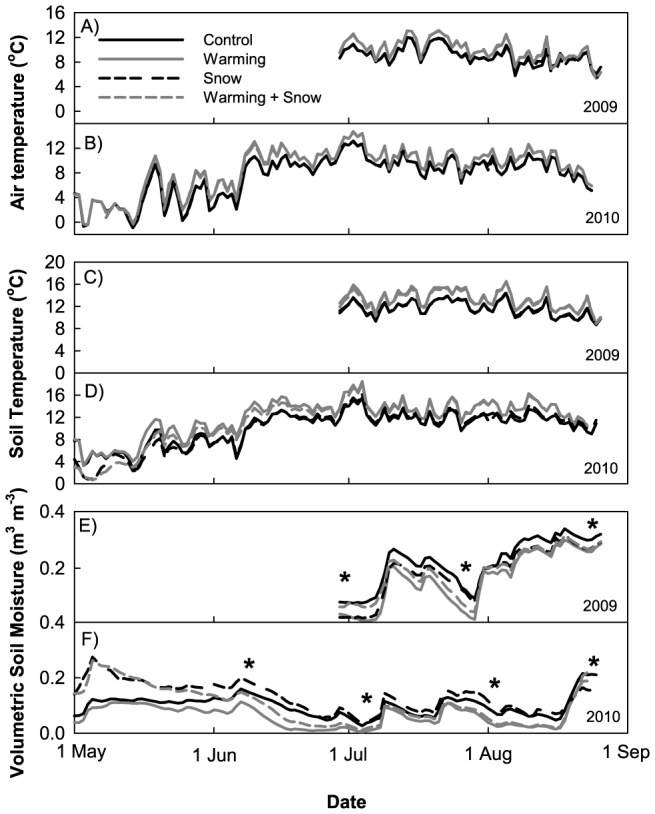
Meteorological measurements. A, B) Air temperature measured at 10 cm above the soil surface for 2009 and 2010. Note that the snow addition control plot line overlays the snow addition plot line, and the warming plot line overlays the warming×snow addition plot line. C, D) Soil temperature integrated from 0-10 cm below the soil surface for 2009 and 2010. E, F) Soil moisture integrated from 0–10 cm below the soil surface for 2009 and 2010. Asterisks represent the days when soil and plant samples were collected for stable isotope analysis.

The growing season of 2009 was cooler than in 2010 (NOAA, Global Historical Climatology Network, Bangoin Station); in 2009, the seasonal average air temperature (29 June to 24 August) was cooler than in 2010 by 0.35°C (C), 0.50°C (W), 0.45°C (S), and 0.53°C (WS). In both 2009 and 2010, daily mean air temperature was significantly different for all treatment pairwise comparisons, except between C and S plots, and between W and WS plots. The mean air temperature for the entire growing season in 2009 was 9.06°C (C), 10.02°C (W), 8.96°C (S), and 10.03°C (WS). The mean air temperature for the entire growing season in 2010 was 9.41°C (C), 10.52°C (W), 9.41°C (S), and 10.56°C (WS) (Figure 2).

The seasonal mean soil temperature in 2009 (29 June to 24 August) was also cooler than 2010 by 0.36°C (C), 0.51°C (W), 0.62°C (S), and 0.54°C (WS). In both 2009 and 2010, there were significant differences in soil temperature between the four treatments (p<0.0001), and across the season (p<0.0001). In 2009, daily mean soil temperature was significantly different in all treatment pairwise comparisons. In 2010, daily mean soil temperature was significantly different in all treatment pairwise comparisons except between C and S plots. The mean soil temperature for the entire growing season in 2009 was 11.60°C (C), 13.28°C (W), 11.72°C (S), and 13.14°C (WS). The mean soil temperature for the entire growing season in 2010 was 11.96°C (C), 13.79°C (W), 12.34°C (S), and 13.68°C (WS).

2009 was a wetter-than average year, while 2010 was a drier-than-average year, compared to the 50-year mean for May through August (NOAA, Global Historical Climatology Network, Bangoin Station). Mean soil moisture (29 June to 24 August) in 2009 was higher than in 2010 by 0.13 m^3^m^−3^ (C), 0.11 m^3^m^−3^ (W), 0.08 m^3^m^−3^ (S), and 0.12 m^3^m^−3^ (WS). In both 2009 and 2010, there were significant differences in soil moisture between the four treatments (P<0.0001), and across the season (P<0.0001). In 2009, soil moisture probes were not installed until late June, and therefore we were unable to capture soil moisture levels during the snowmelt period. In 2009, soil moisture levels were low until monsoon rains arrived in mid-July (Figure 2) and the highest soil moisture levels did not occur until late August. In 2010, we were able to capture soil moisture during the snowmelt period. During early May 2010, the S and WS plots had the highest soil moisture levels (Figure 2). Around 21 May, however, soil moisture in the WS plots began to decrease while soil moisture in the S plots remained level. Similarly, soil moisture in the W plots began to decrease while the soil moisture in the C plots remained level.

### Source water, soil, and plant water isotopes

There were three sources of water the plants could use at the site: ground water, snowmelt, monsoon rains. Groundwater δD was highly consistent across the season and between years. Seasonal ground water δD was −130.1±0.21‰ (2009) and −129.7±0.26‰ (2010). In 2009, the snowmelt isotope signature could not be used, as the samples were not kept frozen and evaporation had occurred. Snowmelt δD in 2010 was −115.4±1.00‰. Rain δD values were usually more enriched at the start of the season and became progressively more depleted as the monsoon rains intensified. From 9 July to 23 August 2009, rain δD values ranged from −114.1‰ to −127.9‰. From 7 June to 25 August 2010, rain δD values ranged from −31.3‰ to −218.6‰. The local meteoric water line (LMWL) from 2009 and 2010 precipitation data was: δD  = 8.76* δ^18^O + 22.79. Average deuterium excess, *d*, was 6.54±0.85‰ (2009) and 16.19±2.15‰ (2010).

**Figure 3:**
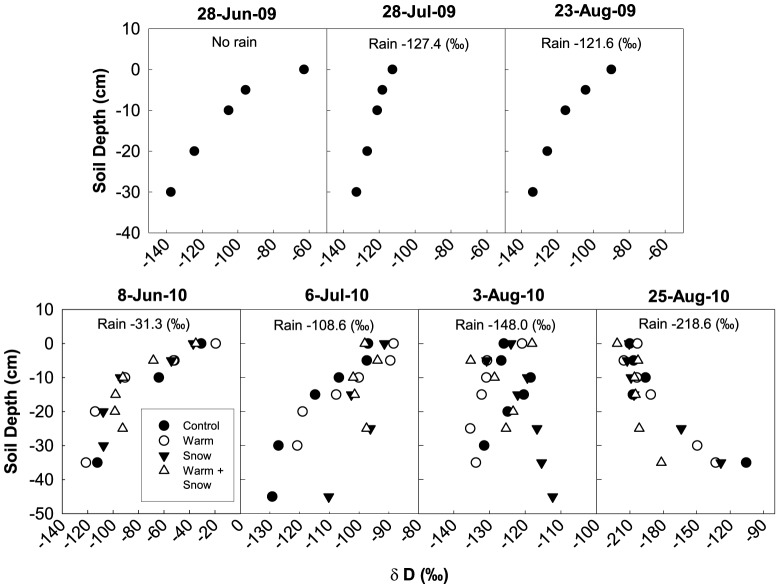
Soil isotope measurements. δD (‰) of soil water at different depths during 2009 and 2010. Rain δD values from those collection periods are also reported.

Without multiple soil core samples for each collection date, our soil water δD results provided us with a pattern of soil water δD, but not a sense of the variation. However, we did find some general patterns from 2009 and 2010 across all treatments. For example, soil water profile δD results suggest that rain events rarely penetrate below 25 cm. In both 2009 and 2010, while the upper 25 cm soil water was influenced by a combination of rain events and evaporative enrichment, the deeper soil water was not. During all three collection dates in 2009 (28 June, 28 July, and 23 August), the δD values of upper soils were more enriched than the soils at lower depths (Figure 3).

In 2010, the soil water isotopic profile on 8 June (pre-monsoon) showed that the upper 10 cm soils were less negative than the deeper soils for all four treatments (Figure 3), suggesting evaporative enrichment. It should be noted that the C plot was missing two soil water values between 10–35 cm depths. The δD values of soils above 10 cm for the next two collection periods of 6 July (early-monsoon) and 3 August (monsoon) showed there was less evaporative enrichment in the upper soil layers. During the last collection on 25 August (late monsoon), there was a large shift in the soil water δD in the upper soils. The upper 15 cm soils became more negative than the deeper soils. The upper soils reflected the very isotopically negative and relatively large rain event that fell on the site just prior to the collections. The deeper soil water in the WS plot became more isotopica, such lly depleted, suggesting that the large rain even had penetrated deeper in soil profile, while in the C, W, and S plots, the deeper soils became more isotopically enriched when compared to 3 August.

**Figure 4:**
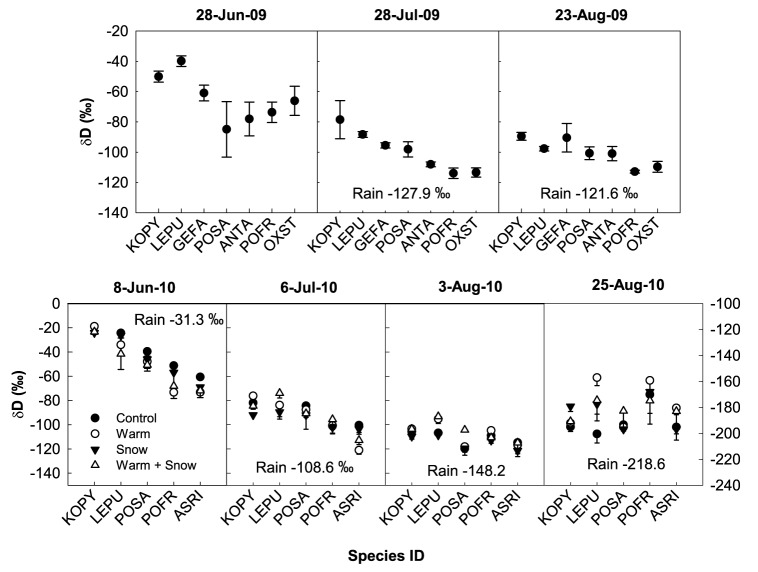
Plant isotope measurements. δD (‰)±1 SE values of plants water collected during 2009 and 2010 (n = 3). Plants are arranged from shallowest roots (left) to deepest roots (right). KOPY = *Kobresia pygmaea*, LEPU = *Leontopodium pusillum*, GEFA = *Gentiana farreri*, POSA = *Potentilla saundersiana*, ANTA = *Adrosace tapete*, POFR = *Potentilla fruticosa*, OXST = *Oxytropis stracheyana,* and ASRI = *Astragalus rigidulus*. For dates 8-Jun-10, 6-Jul-10, and 3-Aug-10, the corresponding y-axis is on the left; for date 25-Aug-10, the corresponding y-axis is on the right. Rain δD values from those collection periods are also reported.

Plant water isotopes in 2009 showed a pattern of water partitioning between shallow and deep soils among the different plant functional groups at the start of the growing season when snowmelt was the dominant water source, but towards the end of the growing season, all plants were using monsoon rain from the upper soil layers as the main water source. During the first collection on 28 June (pre-monsoon), *L. pussilum*, a shallow-rooted species, had significantly less negative δD values than *P. saundersiana*, a deeper-rooted species (P = 0.03)(Figure 4), suggesting that *L. pussilum* was accessing water from the upper soils, while *P. saundersiana* was accessing water from deeper soils (See Table 1 for all pairwise comparisons). On 28 July (monsoon), there was similar partitioning of soil water use between the shallow and deep-rooted species, although all the species were beginning to use more isotopically negative rainwater. By 23 August (late monsoon), all the plant species were using more isotopically negative rainwater, although the δD value of deep-rooted *P. fruticosa* was still significantly different from shallow-rooted *K. pygmaea* (P = 0.04) and *L. pussilum* (P = 0.04).

**Table 1:** Pairwise comparisons of different plant species and treatment δD (‰) for 2009 and 2010.

28-Jun-2009	28-Jul-2009	23-Aug-2009	
LEPU vs. POSA **	KOPY vs ANTA *	KOPY vs. POFR *	
	KOPY vs. OXST **	LEPU vs. POFR *	
	KOPY vs. POFR **		
	LEPU vs. POFR *		
	LEPU vs. OXST *		
			
**8 -Jun-2010**	**9-Jul-2010**	**3-Aug-2010**	**25-Aug-2010**
KOPY vs. POSA **	KOPY vs. POFR **	KOPY vs. ASRI *	POSA vs. POFR *
KOPY vs. POFR **	KOPY vs. ASRI **	LEPU vs. POSA **	
KOPY vs. ASRI **	LEPU vs. POSA **	LEPU vs. ASRI **	
LEPU vs. POFR **	LEPU vs. POFR *		
LEPU vs. ASRI **	LEPU vs. ASRI **	S vs. W *	
POSA vs. ASRI **		S vs. WS *	
POSA vs. POFR **			
			
C vs WS *			

There were no treatments in 2009. Non-statistically significant comparisons were not included. Rooting depths from shallow to deep are as follows (5–35 cm): *Kobresia pygmaea* (KOPY), *Leontopodium pusillum* (LEPU), *Potentilla saundersiana* (POSA), *Potentilla fruticosa* (POFR), *Oxygropis stracheyana* (OXST), *Astragalus rigidulus* (ASRI). The four treatments were: C = control, W = warming, S = snow addition, WS = warming×snow addition. * signifies P<0.05, ** signifies P<0.005.

In 2010, we analyzed differences in plant water δD among the four sampling periods, the four treatments, and plant species. We found a significant difference in plant water δD for date (P<0.001), and species (P<0.001), but not for treatment; however, there was a significant interaction between date * treatment (P = 0.02), and date * species (P<0.001). Because of this significant interaction involving date, we then ran a two-way ANOVA for each of the four sampling dates separately, in order to test for differences in species and treatment within each sampling period.

Similar to our findings from 2009, in 2010 we found water partitioning at the start of the growing season, but by the end of the growing season, all the plants were using rainwater from the upper soil layers. On 8 June (pre-monsoon), we found many significant differences in plant water δD among the shallow-rooted versus deep-rooted species (see Table 1 for all pairwise comparisons). On 6 July (early-monsoon), again we found that shallow-rooted species *K. pygmaea* and *L. pussilum* had significantly less negative δD than deep-rooted *P. fruticosa* and *A. rigidulus*. Once the monsoon rains had begun by 3 August, we saw fewer differences in δD between shallow- and deep-rooted species, and by 26 August (late monsoon), almost all the species were using primarily rain water from upper soils (Figure 4).

In 2010, the treatments had an overall effect on plant water δD during two of the four sampling periods. On 8 June (pre-monsoon), the C plot was significantly less negative than the WS plots (P = 0.05), indicating that plants in the WS were taking up the more depleted snow as a water source. On 3 August (monsoon), the S plots were significantly more negative than the W plots (p = 0.05) and WS addition plots (P = 0.05), indicating that plants in the warmed plots were using more isotopically enriched water.

### CO_2_ flux measurements

**Figure 5:**
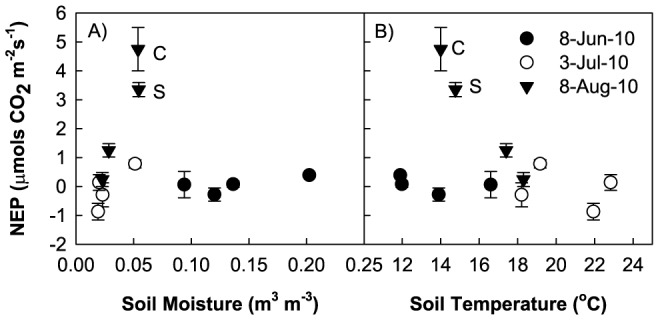
Net Ecosystem Productivity. Relationship between: (a) NEP and soil moisture and (b) NEP and soil temperature, during the three periods when CO_2_ flux measurements were made during the 2010 growing season: 8 June (pre-monsoon), 3 July (pre-monsoon), and 8 August (monsoon). C = control, S = snow addition.

During the three periods of CO_2_ flux measurements in 2010, there were differences in soil moisture, soil temperature, and photosynthetically active radiation (PAR) levels among the treatments and across the growing season. We omitted air temperature from the analysis since air and soil temperature were highly correlated (R^2^ = 0.91). Multiple regression analysis found that all three environmental variables were statistically significant in accounting for differences in NEP (P<0.001; NEP = 2.356–16.473*(soil moisture) - 0.202*(soil temperature) + 0.00141*(PAR), R^2 = ^0.2). Although this regression was statistically significant, the low R^2^ value prompted us to further investigate the negative relationship between NEP and both soil moisture and soil temperature. We plotted NEP against soil moisture and NEP against soil temperature, but grouped the results by the period when NEP measurements were made (i.e. 8 June, 7 July, and 8 August). We found that two data points drove the negative relationship between NEP and soil moisture, and NEP and soil temperature: C and S plots from the 8 August collection (Figure 5A, B, triangle symbols)

**Figure 6:**
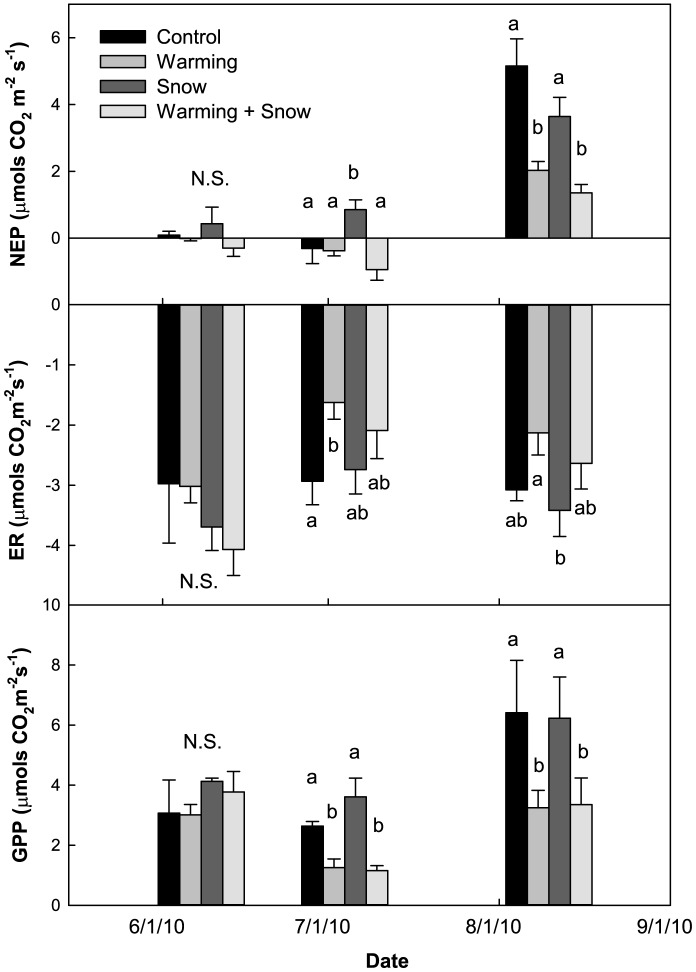
CO_2_ flux measurements. Net Primary Productivity (NEP), Ecosystem Respiration (ER), and Gross Primary Productivity (GPP) measured at midday (11:00–13:00) at three different periods during the growing season. The different letters signify statistically significant differences (P<0.05) in the CO_2_ fluxes, N.S. = not statistically significant.

Within each collection date, we analyzed for differences in NEP, ER, and GPP among the four different treatments (Figure 6). On 8 June (pre-monsoon), we did not find any significant differences in NEP, ER, or GPP among the four treatments. However, on 3 July (pre-monsoon), we found that S plots had significantly higher NEP than the other three treatments and this was largely due to higher GPP rates in S plots than in W (P = 0.002) and WS plots (P = 0.001). GPP in C plots was also significantly higher than W (P = 0.03) and WS plots (P = 0.02), but the high ER rates in C plots resulted in no statistical difference in NEP rates between C and W plots, or C and WS plots. On 8 August (monsoon), we saw the highest rates of NEP. NEP in the C and S plots was significantly higher than W and WS plots, and this was largely due to higher GPP in the C and S plots.

## Discussion

Rain is the dominant precipitation type on the TP and our study found that all the plants, regardless of rooting depth, relied on rain as the main water source in the grasslands in the Nam Tso region. The five main conclusions of our study were: 1) summer monsoon rains originate from two sources: the East Asian Monsoon and from local convective storms, 2) plants partitioned water use pre-monsoon, but during the monsoon, all plants used mainly rain from the upper soil layers, 3) snow additions did not mitigate the effects of the warming treatments by increasing soil moisture or decreasing air and soil temperatures during peak growing season, 4) high NEP rates occurred when the plants were using predominantly summer monsoon rains, and 5) the TP is unique from other alpine ecosystems because rain, not snow, drives productivity in this system.

### Summer rains originate from two sources

The relationship between δD and δ^18^O of precipitation throughout the growing season demonstrate that summer monsoon rains originate from two sources: the Indian Ocean and local convective storms. First, isotopically lighter summer rains compared to winter snow demonstrate that monsoon rains originated from the Indian Ocean. The more negative δD and δ^18^O rain values are a result of rain-out depletion of heavier isotopes; the storms originating from the Indian Ocean must travel to very high altitudes with very low temperatures before reaching the TP [7], [29]. Our constructed local meteoric water line (LMWL) was also almost identical to one from Keil et al. [34], where they also found the monsoonal rain atmospheric moisture at Nam Two originated from the South Indian Ocean. Second, *d*-excess calculations provide evidence that some storms were local, convective storms. The *d* of the Global Meteoric Water Line (GMWL) is 10‰ [29], and values greater than 10 suggest a moisture source from evaporated water fluxes over continental land surfaces [35], [36]. Our site was close to Nam Tso Lake, which is the second largest saline lake on the TP and was most likely the source of moisture for local storms. In 2009, *d* = 6.43‰, while in 2010, *d* = 16.19‰. This suggests that more convective storms occurred during 2010 than in 2009. However, the monsoon season on the TP can extend well into September, and our last collection in 2009 was on 23 August. Rain collected after 23 August may have revealed more rain originating from convective storms. Our findings are also consistent with those from Xu et al. [37], where they found evaporated lake water to contribute to summer precipitation. As lake levels rise in response to climate [38], this could have important feedbacks to local precipitation and local moisture availability.

### Temporal variation in water use partitioning by different plant functional groups

In many ecosystems, water resource partitioning by different plant functional groups allows different species to co-exist [8], [39]–[42]. One classic example of this partitioning is in grasslands, where shallower-rooted grasses tend to use water from the upper soil layers, while deeper-rooted shrubs use water from deeper soil layers. In our study, we did find water partitioning between the shallow and deep-rooted plants, but only during periods that precede the monsoon rains. During the monsoon season, we saw less water use partitioning, and by late August in both years, all plants were using water from the upper soils, which were dominated by rain.

Although many studies have found distinct partitioning in water use between shallow- and deep-rooted species, some studies have also found deep-rooted species to occasionally use water from the upper soils, which are dominated by rain [40], [43]. These studies found that deeper-rooted species only used rainwater late in the growing season when the deeper soils were extremely dry. In our study, we also found that deeper-rooted plants used water from upper soil water late in the growing season. This pattern of water use was likely due to the coupling between water and nutrient availability at our site. In many ecosystems, nitrogen availability is tightly linked to water availability, [44]–[46] and in many tundra systems, nitrogen availability is linked to snowmelt [47], [48]. However, because monsoon rains are the main precipitation type on the TP, the timing of nitrogen availability may be linked to the arrival of monsoon rains. Since nutrients are usually concentrated in the shallow soil layers [49], [50], the deeper-rooted plants may be using moisture from the shallow soil layers during the monsoon period to access nutrients. Deeper-rooted plants may also have increased fine root biomass in the upper soils during the monsoon season in order to access the nutrients, thereby increasing water uptake from the upper soils.

### Snow additions did not mitigate warming effects

While snowmelt increased soil moisture at the beginning of the growing season, the extra snow did not increase soil moisture levels in WS plots or decrease air and soil temperatures in WS plots for the entire summer. By the end of June in both years, WS plots had the same levels as W plots. Similarly, in both years, air and soil temperature averaged over the growing season in WS plots was identical to W plots.

Snow addition played an important role in determining plant water δD at the start of the growing season, but warming had a much larger effect on plant water δD towards the end of the growing season. For example, on 8 June 2010 (pre-monsoon), plants in the S and WS plots had more negative δD than plants from the C plots (Figure 3) because more isotopically depleted snow (−115‰) was added to S and WS plots, suggesting that the plants were, in fact, utilizing this extra pulse of moisture from snow at this time. However, by 3 August (monsoon), rain was the dominant water source used by all the plants, and warming became a more important factor influencing plant water δD. For example, on 3 August, plants in the S plots had significantly less negative values than plants in the W plots. This pattern was probably due to an earlier dry-down of upper soils in the W plots. When the rains arrived, the upper soils in W plots became saturated with rainwater (with a more negative δD value of −148‰), while the upper soils of S plots had a mixture of rain and residual soil water, which retained some of the more isotopically less negative snow melt.

Historically, the TP does not have a persistent standing snowpack, and snow that falls either quickly melts or sublimates. In our study, we found that precipitation inputs from winter/spring was pushed down from shallow soils to deeper soil layers as soon as the monsoon rains arrived. For example, in 2010, during the early part of the growing season, the δD of soil water above 20 cm reflected a pattern of evaporative enrichment (Figure 3); however, once the monsoon rains arrived, the upper soil reflected a more isotopically depleted monsoon rain signal while pushing the isotopically enriched upper soil water into deeper soil layers (Figure 3, particularly C and W plots). During the monsoon season, we also found that all plants were accessing water from the upper soils, which were dominated by rain. Therefore, the extra snowmelt was not being utilized once the rains arrived. These results also suggest a large disadvantage for the shallow-rooted species, such as *K. pygmaea*, under warmer conditions. Warming drastically reduced soil moisture levels pre-monsoon (Figure 2) and caused higher evaporative enrichment in the soil water δD (Figure 3), and this led to *K. pygmaea* senescing earlier in the W plots (data not presented in this paper). During this period, the deeper-rooted species, such as *O. stracheyana* and *P. fruticosa*, were able to access water deeper in the soils. Once the upper soils dried, *K. pygmaea* had no other water source and quickly senesced. Zhang and Welker [51] found another species of *Kobresia* on the TP, *Kobresia humilis*, to have very low nutrient and water uptake rates. They found that while warming stimulated aboveground productivity in many graminoid species, *K. humilis* had no response and could not compete with grasses for nutrients. In Tibet's alpine meadows, *K. pygmaea* covers up to 90% of the landscape [52] and is the dominant summer grazing vegetation for yaks in this region. Increases in snow at the start of summer did not appear to benefit *K. pygmaea*, and continued warming may cause decreases in *K. pygmaea* across the landscape.

### High rates of NEP coincide with the arrival of monsoon rains

Snow addition did not dramatically increase ecosystem NEP during the early part of the growing season. On 3 July (pre-monsoon), we did find that NEP for S plots was significantly higher than the other three treatments, but NEP rates during this period were also relatively low compared to the 8 August (monsoon) measurements. By 8 August, snow addition had no effect on NEP, since S and C plots both had high NEP rates. Warming greatly reduced NEP, even in WS plots. By the end of June, WS plots had soil moisture levels similar to that of W plots, despite the extra snow input at the start of the growing season. These results suggest that increases in snowstorms may not alleviate water stress under a warmer climate. While increases in summer monsoon rains may help alleviate water stress, Wu and Qian [53] found a link between snowfall and rain, where years with high snowfall during winter were correlated with years with less rain during the summer. A winter with large snowstorms followed by a drier summer could be detrimental to shallow-rooted plants and also alter carbon uptake in this ecosystem.

In the past, studies have found a positive relationship between NEP and soil moisture [44], [54]–[56], and a negative relationship between NEP and soil temperature [20], [57], [58]. We found both relationships to be negative; however, two collection points largely drove the negative relationship: the C and S plot measurements during the 8 August (monsoon) collection. This suggests that soil moisture and temperature limited NEP during different times of the growing season. On 8 June (pre-monsoon), NEP was low for all treatments, despite some of the wettest soils, suggesting that low soil temperatures limited NEP (Figure 5). On 3 July (pre-monsoon), again all four treatments had relatively low NEP. However, during this period, soil moisture was the lowest and soil temperature was highest, suggesting that both dry and warm soils limited NEP. This is consistent with other studies that find high soil temperatures reduce photosynthetic uptake by plants by inducing water stress [59], [60]. On 8 August (monsoon), soil moisture was slightly higher than on 3 July and soil temperatures were cooler than 3 July, but warmer than 8 June, resulting in the highest measured NEP. This suggests that during the 8 August collection, soil moisture and temperature were at optimal conditions to favor high NEP. By 8 August, we did see an increase in biomass in all the plots (data not presented in this paper), which would lead to higher GPP rates, and ultimately higher NEP [44]. However, we still found a difference in NEP between the warmed and non-warmed plots, which again suggests that soil temperature and moisture play important roles in driving NEP.

### The TP is unique from other high-elevation ecosystems

Unlike other alpine ecosystems, rain is the dominant precipitation type on the TP, and this difference between dominant precipitation types has implications for the timing of maximum productivity. In most alpine ecosystems, soil moisture reaches a maximum following snowmelt. Productivity is tightly coupled to soil moisture [61]–[63] and because of the relatively short growing season length in alpine ecosystems, the highest rates of productivity typically occur during the early part of the growing season [64]. In ecosystem warming experiments that are snowmelt dominated, productivity rates usually occur during early summer, but then productivity decreases when soils begin to dry due to warming [60]. Larger snow packs in these systems would keep the soils moist longer throughout the season and potentially reduce water stress in the plants, though they may also inhibit other developmental processes [65]. Contrary to other alpine ecosystems, on the TP, maximum soil moisture levels occur during the middle of the growing season, following monsoon rains. We found that an increase in soil moisture levels at the start of the growing season could not alleviate warming-induced water stress later in the growing season because the snowmelt water moved quickly through the soil profile.

There are three possible explanations for why shallow-rooted plants in our experiment did not take advantage of this extra snowmelt earlier in the season. First, for many plants, the “turn on” for spring involves multiple environmental stimuli, including adequate day length, soil temperatures, and soil moisture [65]–[67]. Our snow addition may have increased soil moisture levels too early in the growing season, when other environmental cues were not at adequate levels to stimulate growth. Second, the timing of maximum soil moisture levels is also linked to the timing of nutrient availability; this in turn affects the timing of nutrient acquisition by plants, and ultimately the timing of high productivity rates. While in many tundra systems, the highest levels of nitrogen availability usually follow snowmelt [68], [69], peak nitrogen availability on the TP may occur later in the growing season, coinciding with the monsoon rains. Third, interspecific differences in plants' ability to respond to soil environment conditions and other phenological cues may drive the ecosystem-level responses we observed. The aboveground biomass is dominated by *K. pygmaea*, a shallow-rooted species that we found to be particularly sensitive to water limitation. *K. pygmaea* may also have conservative growth strategies to protect it from variable environmental stresses early in the growing season. If the timing of *K. pygmaea*'s peak activity has evolved to synchronize with the monsoon, when water stress is less likely, the life history of this single species could be driving much of the ecosystem NEP response that we observed. This explanation is further supported by NDVI data [34] and eddy flux measurements [70] from this region, which indicates that GPP peaks during August, concurrent with the monsoon rains.

One important consideration from our findings is that we are reporting results from only one year of experimental manipulations, although Walker *et al.* [27], used a meta-analysis to show that changes, such as species composition and diversity occurred rapidly even after two years of experimental warming. Other studies in tundra ecosystems have found differences between short-term versus long-term responses [71]–[73]. As plants begin to acclimate to warmer temperatures and increases in spring snow, we may begin to see snowmelt being utilized by some of the plants earlier in the growing season, which in turn may lead to changes in NEP across the landscape. For example, after two years of experimental warming, we found that *K. pygmaea* senesced earlier in the warmed plots. However, Yang et al. [74] found that after four years of experimental warming, *K. pygmaea* adjusted its non-structural carbohydrate accumulation, suggesting that *K. pgymaea* could potentially adjust to warming. Continued monitoring of species- and plot-level responses are needed to further understand the long-term effects of climate change on grassland processes.
